# Temporal, Spatial and Prey Niche Partitioning Reveals Coexistence Mechanism of Mesocarnivores in Guangdong Province, South China

**DOI:** 10.1002/ece3.71797

**Published:** 2025-07-13

**Authors:** Fei Wu, Yan Hua, Hongliang Dou, Yulin Zhang, Xiaoxin Dong, Di Zhu, Haiyang Gao

**Affiliations:** ^1^ Guangdong Provincial Key Laboratory of Silviculture, Protection and Utilization Guangdong Academy of Forestry Guangzhou China; ^2^ National Forestry and Grassland Administration Key Laboratory for Conservation Ecology in the Northeast Tiger and Leopard National Park College of Life Sciences, Beijing Normal University Beijing China

**Keywords:** camera trap, ecological niche, mesocarnivores, species coexistence, wildlife management

## Abstract

Mesocarnivores are expected to maintain ecosystem function and stability as emerging apex predators after the decline and extinction of large carnivores. However, we still do not fully understand the mechanisms by which sympatrically distributed mesocarnivores coexist. Niche partitioning provides a fundamental explanation, yet most studies focus on only temporal and spatial dimensions, often overlooking the role of prey resources. To comprehensively understand mesocarnivore coexistence in the context of widespread large carnivore extirpation, we collected camera trap data from four national and sixteen provincial nature reserves in Guangdong Province, South China. We examined the niche partitioning of four mesocarnivores, including leopard cat ( *Prionailurus bengalensis* ), masked palm civet ( *Paguma larvata* ), spotted linsang ( *Prionodon pardicolor* ), and ferret badger ( *Melogale moschata* ) across temporal, spatial, and prey resources (murids and three pheasant species) dimensions. Results indicate that mesocarnivores exhibit greater degrees of overlap in temporal niche (Dhat4 index ranges from 0.66 to 0.93), compared to spatial niche (Pianka's O index ranges from 0.03 to 0.44). Prey availability is positively associated with mesocarnivores' RAI (relative abundance index), with the RAI of murids serving as a primary explanatory variable for masked palm civet, spotted linsang, and ferret badger's RAI. The abundance of leopard cat is positively associated with the RAI of silver pheasant ( *Lophura nycthemera* ). Abundances of masked palm civet and ferret badger show species‐specific associations with the RAI of white‐necklaced partridge ( *Arborophila gingica* ) and Chinese bamboo partridge ( *Bambusicola thoracicus* ), respectively. Additionally, the spatiotemporal niche overlap among mesocarnivores remains stable between dry and rainy seasons. We conclude that spatial niche partitioning and diverse utilization patterns of prey resources are likely to facilitate the coexistence of mesocarnivores. Our findings provide a multidimensional perspective on mesocarnivore coexistence mechanisms, offering new insights into their ecological interactions and a reference for the conservation and management of mesocarnivores in Guangdong Province, South China.

## Graphical abstract

Through examining the three ecological niche dimensions, we found spatial and prey niche partitioning could help explain mesocarnivore coexistence patterns in Guangdong Province, southern China.

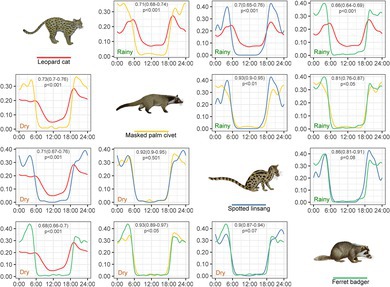

## 1 Introduction

Apex predators like large carnivores commonly occupy the top of the food chain and play a crucial role in maintaining ecosystem function and stability through trophic cascade effects (Paine 1966; Ritchie and Johnson 2009). However, large carnivores are particularly vulnerable to anthropogenic threats, such as poaching and habitat destruction, leading to a rapid decline in populations globally over the past few decades (Corlett 2007; Treves and Karanth 2003). In many regions, large carnivores are undergoing local extinctions and resulting in the ecological release of mesocarnivores (1–15 kg in body mass), which subsequently assume the role of apex predators in local communities. This is a phenomenon known as “mesocarnivore release” (Ripple et al. 2014; Prugh et al. 2009; Wallach et al. 2015). Mesocarnivores facilitate energy flow in the food web by controlling prey populations and contributing to trophic interactions, partially replacing the functions of extinct large carnivores (Avrin et al. 2023). Therefore, mesocarnivores are more likely to play a greater ecological role in the future, yet the coexistence mechanism of mesocarnivores remains unclear (Roemer et al. 2009).

According to the classical competitive exclusion principle, sympatric mesocarnivores must partition at least one niche dimension; otherwise, species with similar niches cannot coexist (Hardin 1960). Therefore, niche partitioning is generally considered a fundamental coexistence mechanism of sympatric species. Closely related species normally partition across multiple niche dimensions within a region (Tian et al. 2022). Temporal niche partitioning, through variations in seasonal or diel activity patterns, allows mesocarnivores to reduce competitive interactions by adopting diurnal or nocturnal foraging strategies (Lee et al. 2024; Rodríguez‐Luna et al. 2024). In the spatial niche dimension, mesocarnivores exhibit distinct spatial distribution patterns, affected by both their specific microhabitat preferences and the heterogeneity of prey resource availability, thereby minimizing spatial niche overlap and reducing interspecific competition (St‐Pierre et al. 2006). In the prey resource niche dimension, competition between mesocarnivores is exacerbated by overlap in prey composition but can be mitigated by reducing reliance on shared prey and increasing prey diversity (Brown et al. 1999; Charnov 1976; Manlick et al. 2017).

Current studies normally focus on only one or two dimensions while overlooking the seasonal variation in prey resource availability, which hinders a comprehensive and multidimensional understanding of mesocarnivore coexistence patterns (Bu et al. 2016; Grabowski et al. 2024; Vilella et al. 2020; Zhao et al. 2020). Although previous studies have provided valuable insights into the spatial and temporal niche partitioning of mesocarnivores in Southwest and North China (Bu et al. 2016; Cong et al. 2024; Zhao et al. 2020), how mesocarnivores coexist in South China after the disappearance of large carnivores remains unclear. In this region, the extirpation of apex predators such as the South China tiger ( *Panthera tigris amoyensis* ), dhole ( *Cuon alpinus* ), and leopard ( *Panthera pardus* ) has led to a transition from the original four‐level to a three‐level ecosystem, reshaping the local vertebrate community structure (Elmshagen et al. 2010; Lau et al. 2010; Roemer et al. 2009; Villar 2023). This provides an opportunity to further explore how mesocarnivores facilitate coexistence through niche partitioning after the loss of large carnivores.

With the camera trap data, this study focused on four mesocarnivores in South China: the leopard cat ( *Prionailurus bengalensis* ), masked palm civet ( *Paguma larvata* ), spotted linsang ( *Prionodon pardicolor* ), and ferret badger ( *Melogale moschata* ). These mesocarnivores exhibit carnivorous or omnivorous foraging strategies (Jennings and Veron 2015; Qian et al. 1976; Wang and Fuller 2003; Xiong et al. 2017), and all of them incorporate birds and small mammals into their daily diets, thus providing a biological basis for investigating niche partitioning based on specific prey resources. We aimed to elucidate the mechanisms facilitating mesocarnivore coexistence by analyzing seasonal niche partitioning across temporal, spatial, and prey resource dimensions and to provide a reference for mesocarnivore conservation and management regionally.

## 2 Materials and Methods

### 2.1 Study Area and Data Acquisition

The study was carried out in Guangdong Province, South China, which is located within the tropical‐subtropical monsoon climate zone. This region features pronounced dry and rainy seasons, with the October‐to‐March dry season receiving 377 mm average annual precipitation, contrasted sharply by the April‐to‐September rainy season's 1431 mm (Ye and Zhang 2023). Due to prolonged anthropogenic disturbances, original evergreen broad‐leaved forests were largely transformed into secondary evergreen needle‐leaved forests, which now only cover 85.21% of the total forest area (Duan et al. 2008; Sun et al. 2013; Li et al. 2024). This large‐scale habitat degeneration, combined with decades of over‐hunting and large predator eradication campaigns between the 1950s and 1960s, has led to the near extinction of large carnivores (Lau et al. 2010; Qin and Nyhus 2018).

**FIGURE 1:**
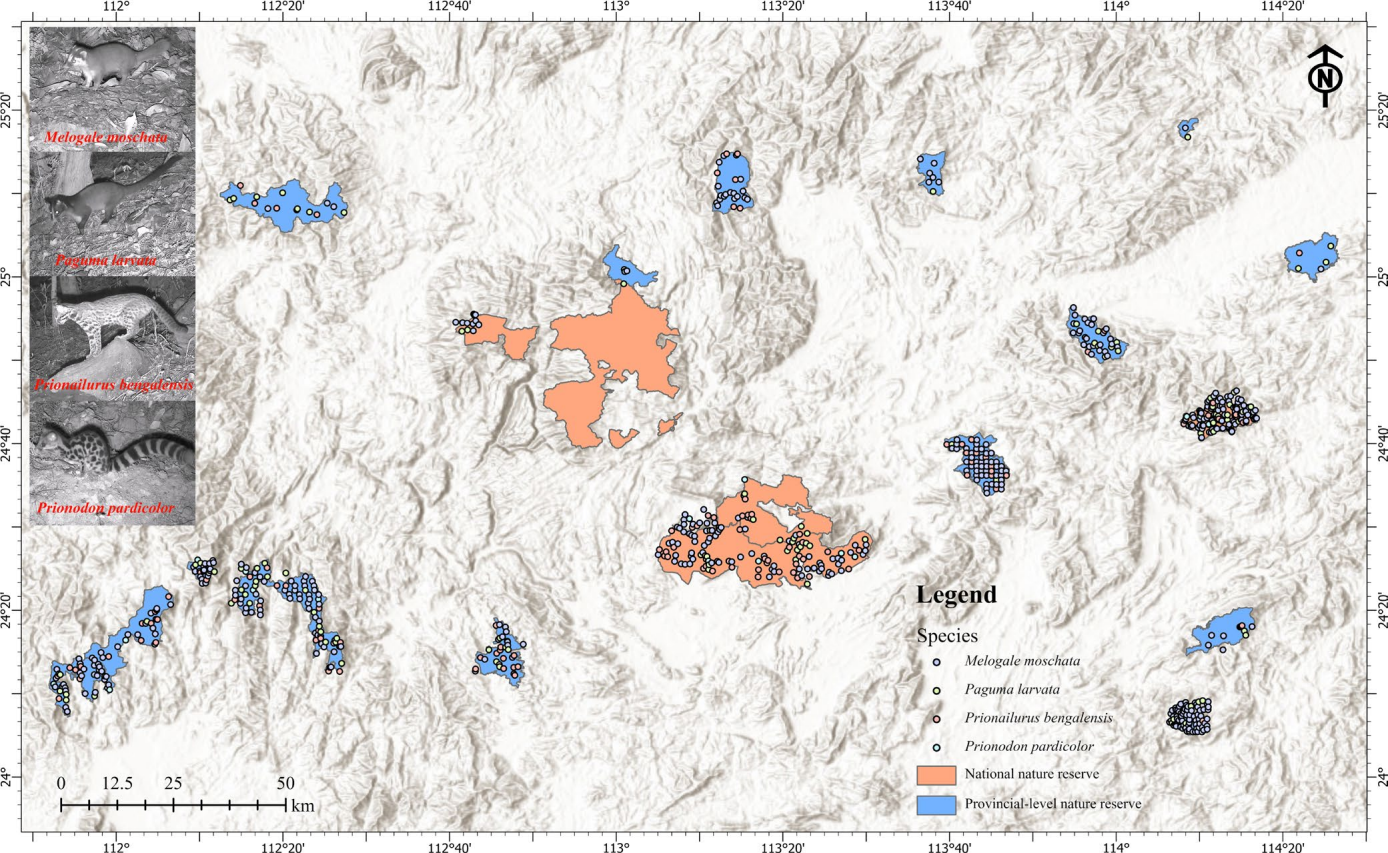
Distribution of four studied mesocarnivores in four national and sixteen provincial nature reserves in Guangdong Province, southern China, with the insets showing the photographs of leopard cat ( *Prionailurus bengalensis* ), masked palm civet ( *Paguma larvata* ), spotted linsang ( *Prionodon pardicolor* ), and ferret badger ( *Melogale moschata* ).

We collected camera trap data from four national and sixteen provincial nature reserves in Guangdong Province, South China, from 2019 to 2023 (Figure 1). The sizes of surveyed reserves range from 1500 to 58,400 ha, and are located in the Nanling Mountain or its southern branches. These reserves encompass forest ecosystems across southern and central subtropical zones, offering high geographical and ecological representativeness. All nature reserves are at the provincial level or higher, with stable financial support, standardized management, and sophisticated camera trap monitoring systems.

A randomized 1 km × 1 km grid design was conducted, and camera traps were deployed following a semi‐standardized sampling protocol, ensuring spatial comparability across different nature reserves while accommodating ecological heterogeneity. Within each grid, cameras were preferentially installed at sites with frequent signs of animal activity, such as trails, water sources, or ridgelines, and properly adjusted based on local topography and vegetation features. Cameras were directly mounted on suitable tree trunks or wooden stakes at approximately 0.6–1.2 m above ground. All cameras operated continuously, 24 h per day, and were programmed to capture three photographs and a 10–15 s video upon each trigger. No bait or extra lure was used during the monitoring period.

In total, we collected data from 1206 camera traps (Table S1). Effective working days of a camera were defined as the number of days between the timestamps of the first and the last capture. After excluding cameras that malfunctioned or recorded fewer than 60 effective days (Kays et al. 2020), 1024 camera traps with a total of 178,227 effective days were retained. We set a minimum 30‐min interval to define independent events, and multiple individuals appearing in the same image were counted as a single independent detection of that species. To minimize statistical errors, only species with more than 200 independent detections were included in the subsequent analyses. After careful screening, four mesocarnivores with body masses ranging from 1 to 15 kg were selected (Prugh et al. 2009), including leopard cat (1099 independent detections), masked palm civet (1314 independent detections), spotted linsang (248 independent detections), and ferret badger (3199 independent detections). Murids and three pheasant species were selected as potential prey for analysis. Due to the small body size and the difficulty of accurate species‐level identification in camera trap images, murids were grouped into a single prey category, with a total of 12,529 independent detections. The three pheasant species were selected based on their shared ecological traits, such as ground foraging and ground nesting behaviors, and their high detection frequencies (each with more than 200 independent detections). These species were the white‐necklaced partridge ( *Arborophila gingica* , 692 independent detections), Chinese bamboo partridge ( *Bambusicola thoracicus* , 732 independent detections), and silver pheasant ( *Lophura nycthemera* , 17,955 independent detections).

### 2.2 Diel Activity Patterns and Temporal Niche Overlap Analysis

We converted the recorded time of all independent detections into solar time to eliminate the influence of region and season variations, using the “overlap” package in R, version 4.3.2 (Nouvellet et al. 2012; R Core Team 2024). The entire day was converted into a range of 0–2π radians, with the time of sunrise and sunset standardized to π/2 and 3π/2, respectively. We defined the crepuscular period as one hour before sunrise to one hour after sunset (Foster et al. 2013).

To analyze the diel activity patterns of mesocarnivores, we used the Kernel Density Estimation (KDE) method from the “activity” package to calculate the overlap of diel activity curves with the Dhat4 index, which ranges from 0 to 1. Values close to 0 indicate complete separation of the temporal niche, while values close to 1 represent complete overlap (Ridout and Linkie 2009). Dhat4 > 0.75 was categorized as a high overlap degree, while Dhat4 < 0.50 indicated a low overlap degree (Marinho et al. 2020). We estimated the 95% confidence intervals (95% CI) of the Dhat4 index using 1000 bootstrap resamples and conducted a randomization test to evaluate the statistical differences in diel activity patterns (Ridout and Linkie 2009; Rowcliffe et al. 2014).

### 2.3 Seasonal RAI Variation and Spatial Niche Overlap Analysis

We calculated the Relative Abundance Index (RAI), defined as the number of independent detection events per 100 camera‐trap days at an observation site for each mesocarnivore species during dry and rainy seasons, at each camera site as well as across the study area. Then we obtained the seasonal RAI from each camera site to assess the degree of spatial niche overlap with Pianka's Overlap Index (Pianka's O) among pairs of mesocarnivore species using the “spaa” package (Mengüllüoğlu and Ambarlı 2019; Pianka 1974; Zhao et al. 2020). Pianka's O ranges from 0 to 1, with values close to 0 indicating high separation and close to 1 representing approximately complete overlap. To better compare the degree of temporal and spatial niche overlap, we used the uniform threshold for assessing temporal niche overlap degree, and we considered Pianka's O > 0.75 as high overlap and Pianka's O < 0.5 as low overlap. We also estimated the 95% CI of Pianka's O using 1000 bootstrap resamples.

### 2.4 Season and Prey Availability Effect Analysis

We tested the effects of season variation and prey availability on mesocarnivores' seasonal RAI using a generalized linear mixed model (GLMM). Because the data distribution of mesocarnivores' RAI is typically left‐skewed and the presence of multiple zero values, we selected “ziGamma” as the distribution family and used the “glmmTMB” package to incorporate a zero‐inflated structure with an intercept term into the regression model, thereby improving the accuracy of parameter estimation (Nooten et al. 2024).

Season (dry and rainy) was included as a categorical fixed effect, along with the seasonal RAI of the three pheasant species and murids (Spearman test, *ρ* < 0.7), and the nature reserve was also included as a random effect. All submodels were compared with Akaike Information Criterion (AIC) values using the “MuMIn” package, and models with ΔAIC ≤ 2 were retained as candidate models (Mazerolle 2006). We maintained the fewest explanatory variables and identified the optimal model among candidate models following the principle of parsimony. To determine the relative importance of each explanatory variable in the optimal model, we conducted a hierarchical partitioning analysis using the “glmm.hp” package to calculate the model contribution rate of each factor (Lai et al. 2022, 2023).

### 2.5 Identifying Spatiotemporal Overlap Difference Between Seasons

To compare seasonal differences, we calculated the centroids of spatiotemporal overlap among mesocarnivores and performed a permutational multivariate analysis of variance (PERMANOVA) based on the Euclidean distance matrix between mesocarnivore species pairs. This method partitions variance in the distance matrix by seasonal variation and fits a linear model. A pseudo‐*F* value was then calculated to evaluate seasonal differences in the spatiotemporal overlap degree and assessed statistical significance via a permutation test with 1000 random permutations. The observed pseudo *F* value was compared with the null model generated by shuffling group labels, ultimately yielding a *p* value that quantifies the likelihood of obtaining the observed differences (Alekseyenko 2016; Anderson and Walsh 2013).

## 3 Results

### 3.1 Diel Activity Patterns and Temporal Niche Overlap Among Mesocarnivores

**FIGURE 2:**
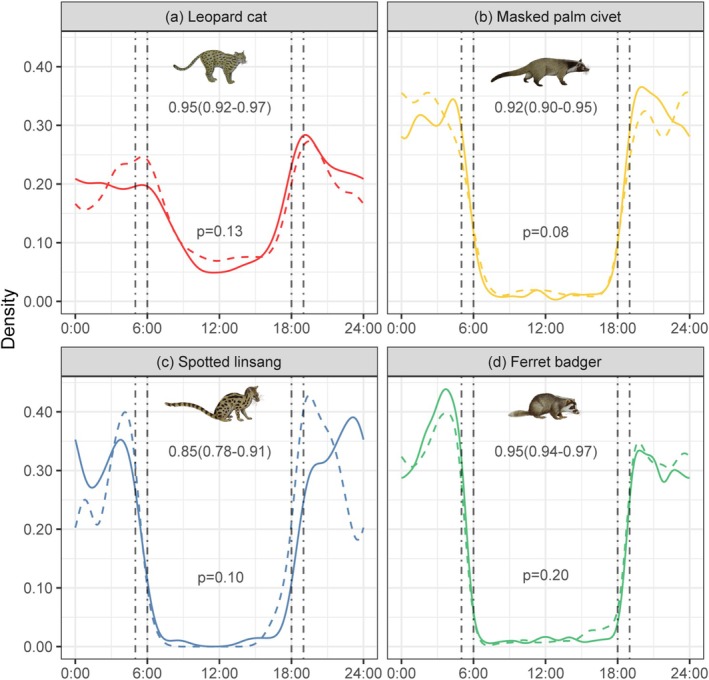
Diel activity patterns of the four mesocarnivores in dry and rainy seasons. The solid line represents the dry season, the dotted line represents the rainy season, and the vertical auxiliary line represents one hour before sunrise or one hour after sunset. Each graph includes a Dhat4 index with 95% CI (top) and the statistical significance of activity patterns (bottom).

**FIGURE 3:**
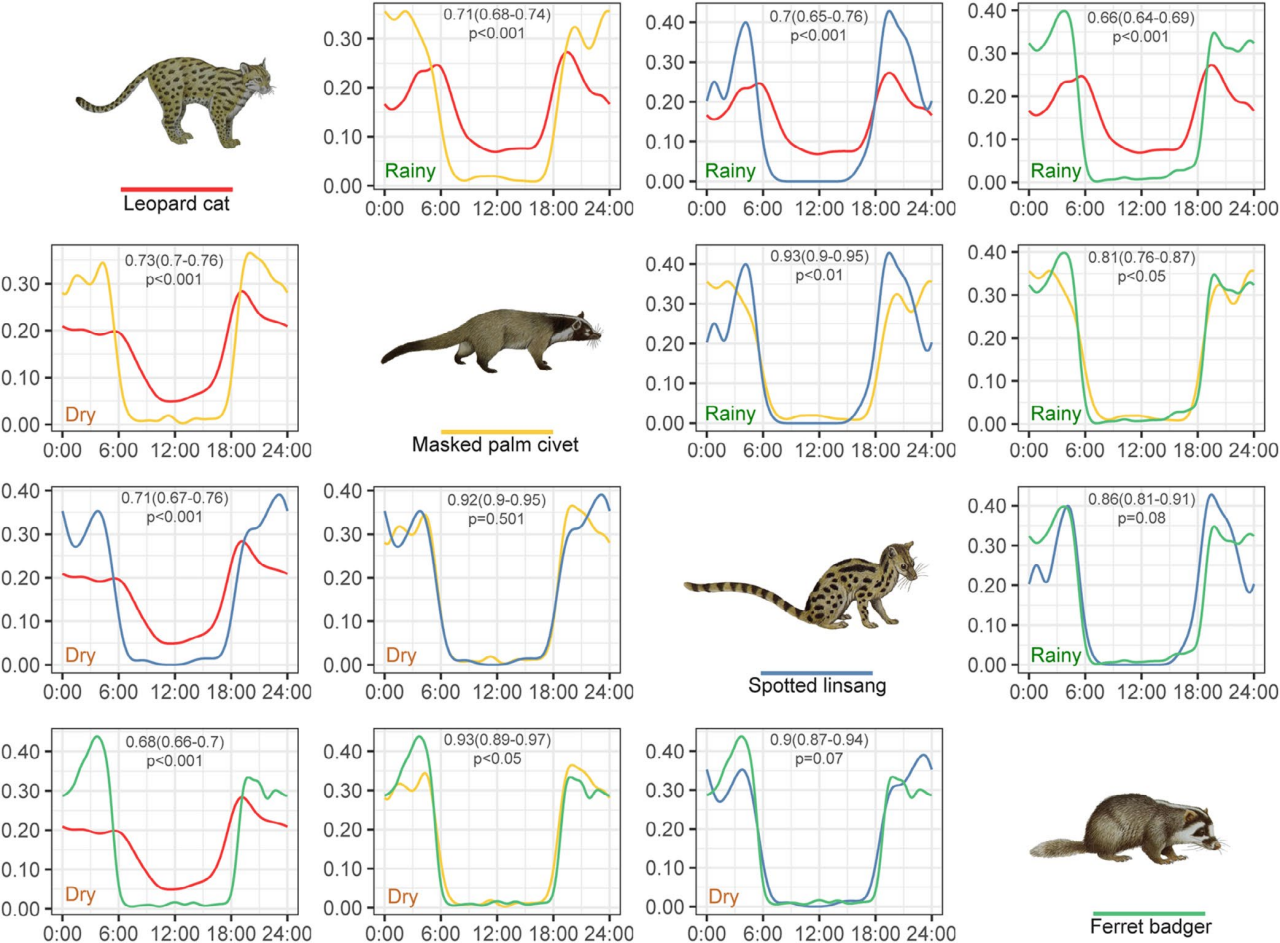
Comparisons of mesocarnivore diel activity patterns in the dry season (left side) and the rainy season (right side). Activity patterns are shown according to species colors (under the silhouette). Each graph includes a Dhat4 index with 95% CI (top) and the statistical significance of activity patterns (bottom).

Diel activity patterns of the four mesocarnivores exhibited a typical bimodal distribution, with peak activity occurring primarily at night (Figure 2), and no significant seasonal differences in activity patterns were detected (randomization test, *p* > 0.05). The temporal niche overlap among mesocarnivores was moderate to high in both the dry and rainy seasons, with the Dhat4 index ranging from 0.66 to 0.93 (Figure 3). Spotted linsang and ferret badger exhibited the lowest overlap degree with leopard cat (Dhat4 = 0.66–0.73), and exhibited a higher overlap degree with masked palm civet (Dhat4 = 0.81–0.93). Only the leopard cat differed significantly from other species in both dry and rainy seasons (randomization test, *p* < 0.001).

### 3.2 Seasonal RAI and Spatial Overlap of Mesocarnivores

**FIGURE 4:**
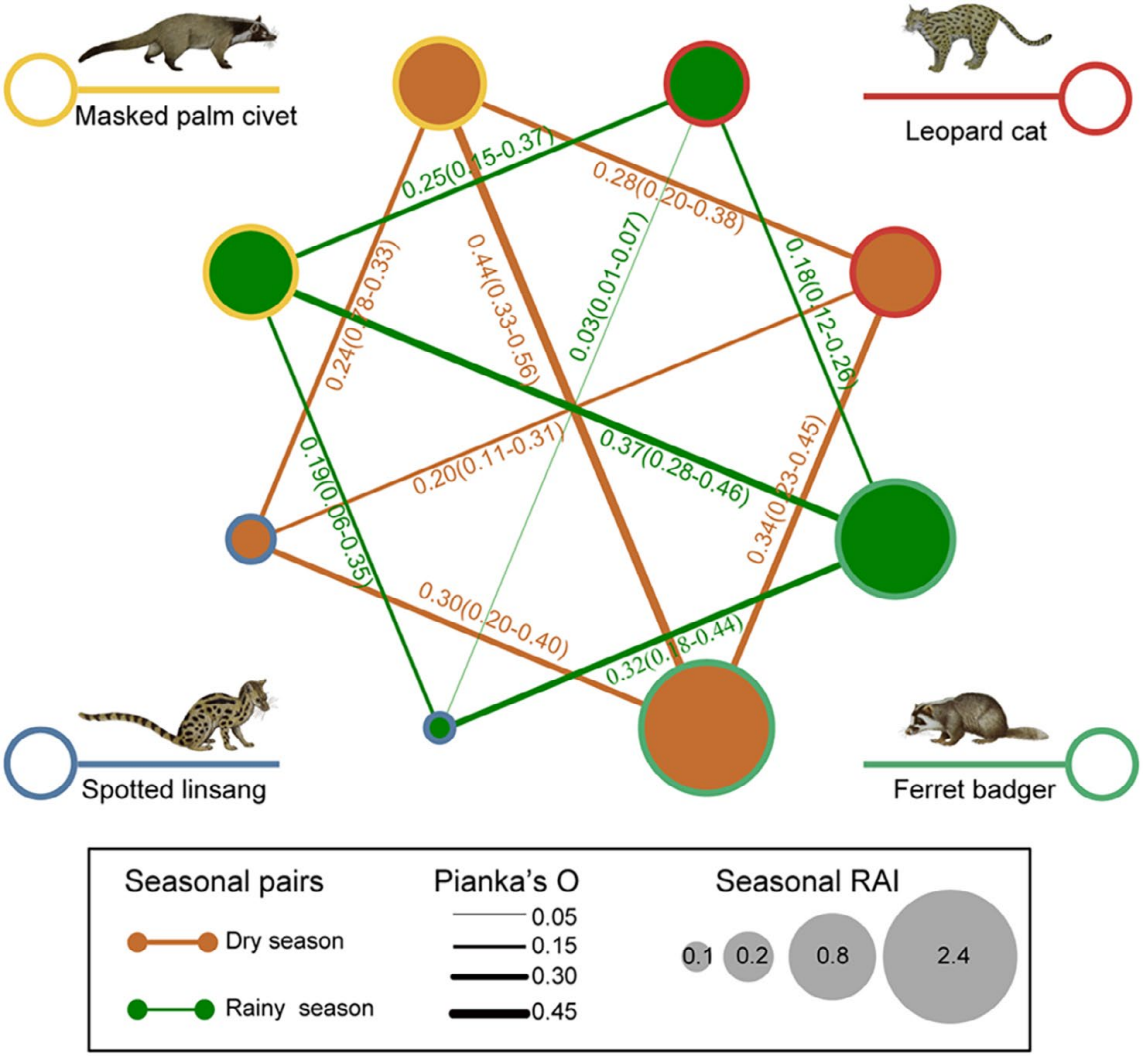
Seasonal RAI and spatial niche overlap degree of mesocarnivore pairs based on Pianka's O index.

Mesocarnivores exhibited a consistent RAI ranking across seasons: ferret badger (dry, 2.16, rainy, 1.47, hereafter), masked palm civet (0.72, 0.75), leopard cat (0.67, 0.57), and spotted linsang (0.19, 0.09) (Figure 4). Three mesocarnivores, including leopard cat, spotted linsang, and ferret badger, had higher RAIs in the dry season than in the rainy season, while the masked palm civet showed an opposite trend.

Spatial niche overlap degree of mesocarnivores was generally low, with Pianka's O index ranging from 0.03 to 0.44 (Figure 4). Except for the spotted linsang and ferret badger pair, spatial niche overlap between other mesocarnivore pairs decreased from the dry to the rainy season. The highest overlap occurs between the masked palm civet and ferret badger in the dry season (Pianka's O = 0.44), while the lowest was between the spotted linsang and leopard cat (Pianka's O = 0.20). In the rainy season, the highest overlap was between the masked palm civet and ferret badger (Pianka's O = 0.37), and the lowest overlap remained between the leopard cat and spotted linsang (Pianka's O = 0.03).

### 3.3 Response of Mesocarnivores' RAI to Season Variation and Prey Availability

**FIGURE 5:**
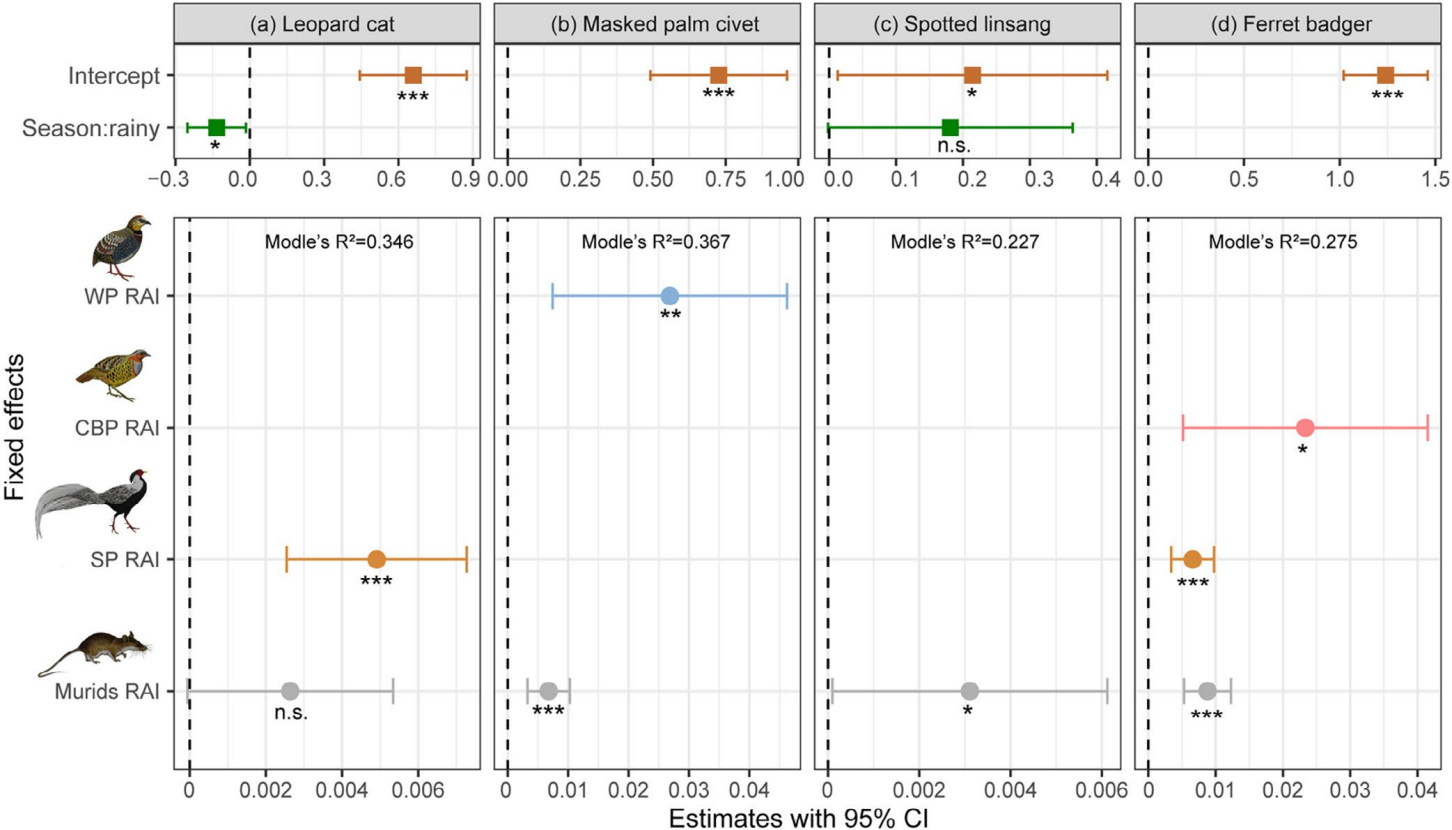
Effects of season variation and prey availability on the relative abundance index (RAI) of four mesocarnivores. CBP, Chinese Bamboo Partridge; SP, Silver Pheasant; WP, White‐necklaced Partridge.

We used season and prey resource availability as fixed effects to construct GLMMs, partially explaining the variation of mesocarnivores' RAI. Among the candidate models with ΔAIC ≤ 2, four models were selected as the optimal explanatory models for each mesocarnivore following the simplicity principle (Table 1). Mesocarnivores RAI showed a positive response to prey resource availability, particularly to murids. Among the four mesocarnivores, only the leopard cat's RAI was significantly affected by seasonal variation, which was significantly lower in the rainy season (*E*_rainy_ = −0.1336, *p* < 0.01, Figure 5).

**TABLE 1:** Generalized linear mixed effect model (GLMM) with ΔAIC ≤ 2 between mesocarnivores' RAI and season variation, prey availability, and variable contribution rate of optimal models according to the simplifying principle.

Species	Modles	Season	WP	CBP	SP	Murids	AIC	ΔAIC ≤ 2
Leopard cat	M1	23.60%			55.16%	21.24%	4066.27	0.00
	M2	+		+	+	+	4067.22	0.96
	M3	+	+		+	+	4067.65	1.38
Masked palm civet	M1		29.80%			70.20%	4116.18	0.00
	M2		+	+	+	+	4117.82	1.63
	M3	+	+			+	4118.01	1.82
	M4		+		+	+	4118.17	1.98
Spotted linsang	M1	+		+		+	1704.15	0.00
	M2	+	+	+		+	1704.45	0.30
	M3	+	+			+	1705.20	1.05
	M4	74.64%				25.36%	1705.28	1.13
	M5	+		+	+	+	1705.35	1.20
	M6	+	+	+	+	+	1705.94	1.79
Ferret badger	M1	+	+	+	+	+	6055.56	0.00
	M2		+	+	+	+	6056.36	0.80
	M3	+		+	+	+	6056.43	0.88
	M4			10.73%	37.25%	50.02%	6056.87	1.31

*Note:* The bolded model represents the optimal model, and the percentage for each fixed effect corresponds to its relative contribution rate.

Abbreviations: CBP, Chinese bamboo partridge; SP, silver pheasant; WP, white‐necklaced partridge.

The responses of mesocarnivores' RAI to different prey RAI varied. The leopard cat's RAI showed a significant positive response to the silver pheasant's RAI (*E* = 0.0049, *p* < 0.001). Masked palm civet's RAI was positively correlated with white‐necklaced partridge's RAI (*E* = 0.2683, *p* < 0.01) and murids' RAI (*E* = 0.0068, *p* < 0.001) with a relative importance (RI) of 70.2%. Murids' RAI had a significant positive effect on spotted linsang's RAI (*E* = 0.043, *p* < 0.05), and the RI of season variation is 45.99%, which was higher than that of murids (RI = 21.12%). The RAI of ferret badger showed a significant positive response to the RAI of murids, Chinese bamboo partridge, and silver pheasant, with murids (*E* = 0.0088, *p* < 0.001) accounting for the highest RI of 47.29% (Figure 5, Table 1).

### 3.4 Spatiotemporal Overlap Difference Between Dry and Rainy Seasons

**FIGURE 6:**
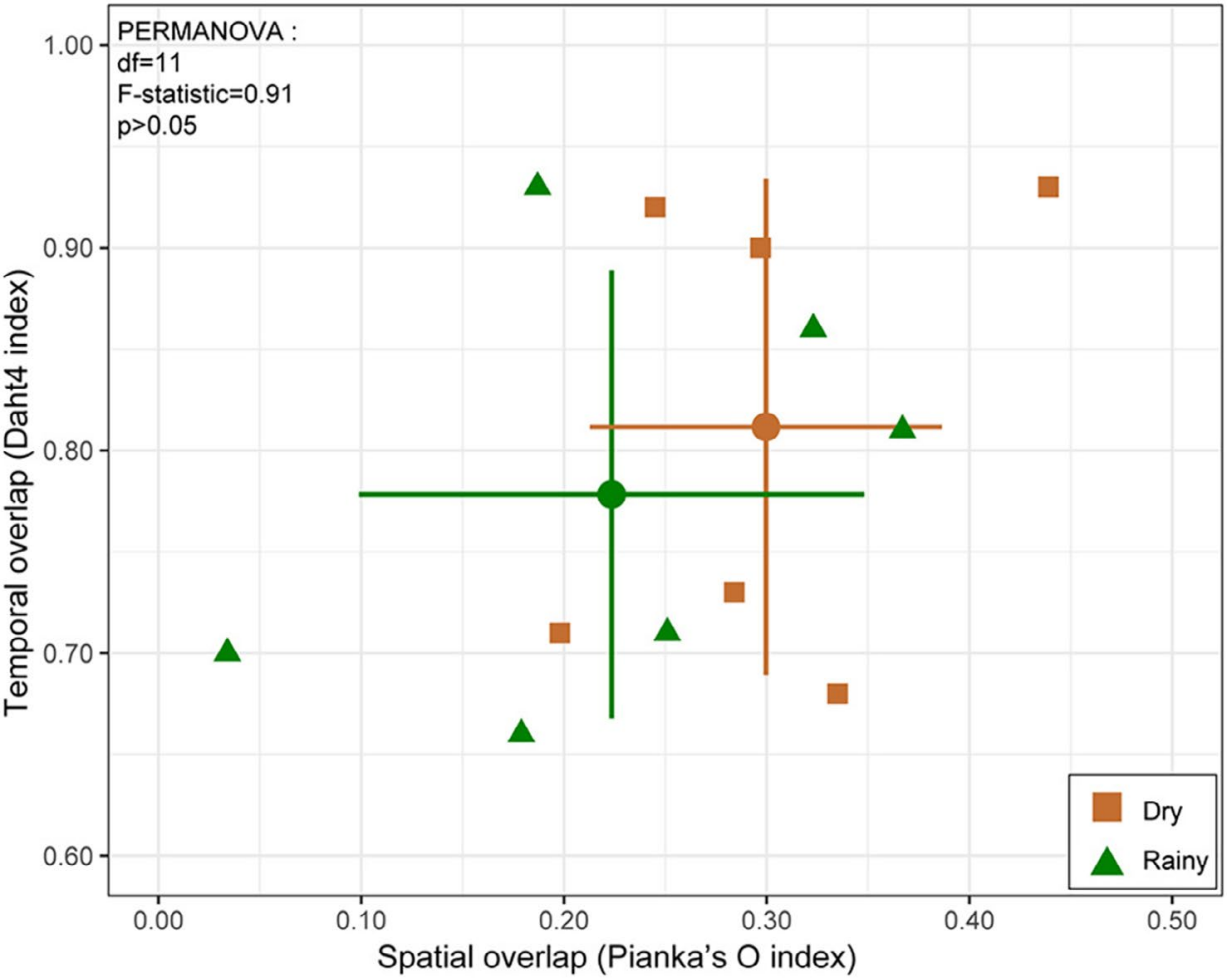
Seasonal temporal overlap (Dhat4 index) and spatial overlap (Pianka's O index) of paired mesocarnivores, and permutational multivariate analysis between dry and rainy seasons. Cross‐shaped error bars indicate the 95% confidence intervals (95% CI), with horizontal bars representing CI for spatial niche overlap and vertical bars for temporal niche overlap.

The average degree of temporal and spatial niche overlap among mesocarnivores was slightly higher in the dry season (Dhat4 = 0.812, Pianka'O = 0.3) than in the rainy season (0.778, 0.224), but the differences were not statistically significant (*F*‐test, *p* > 0.05) (Figure 6). Therefore, seasonal variation has minimal effect on spatiotemporal niche overlap among mesocarnivores.

## 4 Discussion

Our results suggest that the degree of spatial niche partitioning is greater than that of temporal niche partitioning among four mesocarnivores. Furthermore, the relative abundance of mesocarnivores is generally positively associated with prey availability, with murids serving as the primary explanatory variable. Notably, mesocarnivores exhibited species‐specific preferences for different pheasant species. Spatio‐temporal niche overlap among mesocarnivores remained stable across seasons. We concluded that spatial niche partitioning and differential responses to specific prey resources could explain mesocarnivore coexistence in South China.

We found a moderate to high degree of temporal niche overlap among mesocarnivores. Higher temporal overlap was primarily observed among masked palm civet, spotted linsang, and ferret badger (Dhat4 = 0.81–0.93), whereas the leopard cat exhibited a lower degree of temporal niche overlap with the other three mesocarnivores (0.66–0.73). This observation could be closely linked to the predominantly nocturnal habit of mesocarnivores (Bu et al. 2023; Hendry et al. 2023; Zhang et al. 2010) and the higher temporal niche plasticity of the leopard cat, whose diel activity pattern could adjust to the activity patterns of prey availability and the presence of competitors (Kamler et al. 2020; Tian et al. 2022; Xiong et al. 2017). In summary, the moderate to high degree of temporal niche overlap indicates that temporal niche partitioning might have a limited effect on facilitating mesocarnivore coexistence.

Compared to the temporal niche, spatial niche partitioning might play a more significant role in facilitating mesocarnivore coexistence, as mesocarnivores exhibit low spatial niche overlap (Pianka's O, 0.03–0.44). Diverse habitat preferences and foraging strategies could explain spatial utilization differences among mesocarnivores (Manlick et al. 2017; Tomassini et al. 2024). For example, the leopard cat prefers to hunt by ambushing prey species and favors habitats with low vegetation density or forest edges where prey resources are abundant (Hendry et al. 2023; Pereira et al. 2024). In contrast, the masked palm civet and spotted linsang prefer forest habitats with intact vertical structure, reflecting their arboreal habit and ability to forage at multiple canopy levels (Bu et al. 2023). The habitat preference of the spotted linsang is more stringent, and this species is mainly found in primary forest or undisturbed forest, which has never been detected in non‐forest habitats (Jennings and Veron 2015). The ferret badger, which is a typical burrowing animal, prefers areas with lower elevations and rich vegetation types (Zhang et al. 2010). In addition, temporal and spatial niches exist in a coordinated relationship, and high overlap of one niche dimension might promote partitioning of the other niche dimensions (Alipio et al. 2024; Tian et al. 2022; Zhao et al. 2020). Therefore, in this region, the low degree of spatial niche overlap may function as a compensatory mechanism for high temporal niche overlap, thereby enabling mesocarnivore coexistence.

In addition to temporal and spatial niche partitioning, differentiated utilization patterns of the common prey resources may also promote the coexistence of mesocarnivores (Bandyopadhyay et al. 2023; Cong et al. 2024; Schoener 1974). Our results showed that the RAI of all mesocarnivores was positively associated with the RAI of prey. This reflects a direct response of mesocarnivores to fluctuations in prey availability and/or an increased spatial overlap with prey habitats, both of which could enhance predation success (Teixeira et al. 2023; Tomassini et al. 2024). Specifically, in contrast with the other three prey resources, murids were the only prey that showed positive associations with all four mesocarnivores, indicating that murids serve as a basic and shared prey resource (Bu et al. 2023; Mengüllüoğlu and Ambarlı 2019; Silmi et al. 2021), and could potentially intensify interspecific competition. The spotted linsang only exhibited a significant exclusive association with murids. The masked palm civet and the ferret badger show species‐specific associations with the white‐necklaced partridge and Chinese bamboo partridge, respectively. In the optimal model for the leopard cat, the RAI of silver pheasants served as a stronger explanatory variable than that of murids, reflecting its flexible foraging strategy and generalist predatory behavior (Chua et al. 2016; Silmi et al. 2021). Overall, the different responses of mesocarnivores to the three pheasant species imply prey resource partitioning, which might alleviate competition over a single prey and promote species coexistence.

Mesocarnivore coexistence within a guild is a trade‐off between interspecific competition and resource availability, with interactions that might increase during periods of resource scarcity (Finnegan et al. 2021; Torretta et al. 2016). However, our results showed no significant seasonal differences in interspecific spatiotemporal niche overlap. This outcome may be attributed to prey resources remaining sufficiently available in our study area to reduce competition, even during the dry season when prey resource scarcity is often assumed. Additionally, the ecosystem may still be in the early stages of recovery following the disappearance of large carnivores, and such ecological and evolutionary processes are more likely to unfold over extended timescales (Crouzeilles et al. 2016; Ripple and Beschta 2012). Consequently, the observed patterns primarily capture the ecological niche characteristics of mesocarnivores at a particular time point along this long‐term trajectory.

However, given the absence of historical data, it is challenging to track temporal changes in multidimensional coexistence or assess prey resource partitioning. Overall, this study offers a critical baseline for future research on community adaptation and ecological evolution. Future studies integrating dietary analyses and long‐term monitoring are therefore essential to better elucidate the mechanism of mesocarnivore coexistence.

## 5 Conclusion

Through examining the temporal, spatial, and prey niches of mesocarnivores, we found that spatial and prey niche partitioning can explain mesocarnivore coexistence patterns in Guangdong Province, South China. In addition, seasonal fluctuations in resources do not appear to significantly affect mesocarnivore coexistence. This may be attributed to lower population densities and high resource availability at the current ecological stage, which likely reduces negative interactions among mesocarnivores. The loss of large carnivores, coupled with biodiversity conservation policies implemented by the government, means the current population of mesocarnivores is gradually growing. Therefore, it is crucial to understand the dynamics of competition and coexistence among these species, as such insights are key to effective wildlife management and conservation. Given that murids are a shared prey resource, we suggest protecting murids as this might promote mesocarnivore recovery. Furthermore, the primary forests in our study area have undergone severe anthropogenic disturbances over the past century and have been largely replaced by natural secondary forests and highly homogenized artificial forests (Duan et al. 2008). Thus, urgent measures are needed to strengthen habitat protection and restoration. Finally, it seems that mesocarnivore adaptation and evolution have not reached a stable and dynamic equilibrium state, highlighting the need for regular assessment to ensure recovery is in a positive direction. This study provides insights into mesocarnivore coexistence mechanisms and provides a foundation for evaluating the long‐term consequences of large carnivores on mesocarnivores in Guangdong Province, South China.

## Author Contributions

**Fei Wu:** formal analysis (lead), methodology (lead), software (lead), visualization (lead), writing – original draft (lead). **Yan Hua:** project administration (lead), resources (lead). **Hongliang Dou:** project administration (equal), resources (equal), supervision (equal). **Yulin Zhang:** visualization (equal). **Xiaoxin Dong:** project administration (equal), supervision (equal). **Di Zhu:** supervision (equal), writing – review and editing (equal). **Haiyang Gao:** conceptualization (lead), data curation (lead), project administration (lead), resources (lead), writing – review and editing (lead).

## Conflicts of Interest

The authors declare no conflicts of interest.

## Supporting information


Data S1. Raw infrared camera monitoring data of twenty provincial or national nature reserves.

**Table S1.** Camera trap deployment and number of used cameras in studied nature reserves.

## Data Availability

The raw data used in this study are all listed in the Supporting Information.

## References

[ece371797-bib-0001] Alekseyenko, A. V. 2016. “Multivariate Welch t‐Test on Distances.” Bioinformatics 32, no. 23: 3552–3558. 10.1093/bioinformatics/btw524. 27515741 PMC5181538

[ece371797-bib-0002] Alipio, C. , M. R. McCullah‐Boozer , C. L. Gaete , and L. K. Hall . 2024. “Spatio‐Temporal Partitioning Between the Endangered San Joaquin Kit Fox and a Novel Mesocarnivore Community in the Urban Environment as Revealed by Camera Traps.” Global Ecology and Conservation 54: e03184. 10.1016/j.gecco.2024.e03184.

[ece371797-bib-0003] Anderson, M. J. , and D. C. I. Walsh . 2013. “PERMANOVA, ANOSIM, and the Mantel Test in the Face of Heterogeneous Dispersions: What Null Hypothesis Are You Testing?” Ecological Monographs 83, no. 4: 557–574. 10.1890/12-2010.1.

[ece371797-bib-0004] Avrin, A. C. , C. E. Pekins , C. C. Wilmers , J. H. Sperry , and M. L. Allen . 2023. “Can a Mesocarnivore Fill the Functional Role of an Apex Predator?” Ecosphere 14, no. 1: e4383. 10.1002/ecs2.4383.

[ece371797-bib-0005] Bandyopadhyay, M. , S. Biswas , T. Dasgupta , and R. Krishnamurthy . 2023. “Patterns of Coexistence Between Two Mesocarnivores in Presence of Anthropogenic Disturbances in Western Himalaya.” Environmental Monitoring and Assessment 195, no. 3: 397. 10.1007/s10661-023-11003-4. 36781547

[ece371797-bib-0006] Brown, J. S. , J. W. Laundre , and M. Gurung . 1999. “The Ecology of Fear: Optimal Foraging, Game Theory, and Trophic Interactions.” Journal of Mammalogy 80, no. 2: 385–399. 10.2307/1383287.

[ece371797-bib-0007] Bu, H. , J. B. Hopkins , S. Li , and D. Wang . 2023. “Seasonal Distribution and Activity Patterns of Mesopredators and Their Prey in Southwest China.” Journal of Mammalogy 104, no. 5: 941–950. 10.1093/jmammal/gyad034.

[ece371797-bib-0008] Bu, H. , F. Wang , W. J. McShea , Z. Lu , D. Wang , and S. Li . 2016. “Spatial Co‐Occurrence and Activity Patterns of Mesocarnivores in the Temperate Forests of Southwest China.” PLoS One 11, no. 10: e0164271. 10.1371/journal.pone.0164271. 27723772 PMC5056745

[ece371797-bib-0009] Charnov, E. L. 1976. “Optimal Foraging, the Marginal Value Theorem.” Theoretical Population Biology 9, no. 2: 129–136. 10.1016/0040-5809(76)90040-X. 1273796

[ece371797-bib-0010] Chua, M. A. H. , N. Sivasothi , and R. Meier . 2016. “Population Density, Spatiotemporal Use and Diet of the Leopard Cat ( *Prionailurus bengalensis* ) in a Human‐Modified Succession Forest Landscape of Singapore.” Mammal Research 61, no. 2: 99–108. 10.1007/s13364-015-0259-4.

[ece371797-bib-0011] Cong, W. , J. Li , C. Hacker , et al. 2024. “Different Coexistence Patterns Between Apex Carnivores and Mesocarnivores Based on Temporal, Spatial, and Dietary Niche Partitioning Analysis in Qilian Mountain National Park, China.” eLife 13: RP90559. 10.7554/eLife.90559.3. 39259595 PMC11390114

[ece371797-bib-0012] Corlett, R. T. 2007. “The Impact of Hunting on the Mammalian Fauna of Tropical Asian Forests.” Biotropica 39, no. 3: 292–303. 10.1111/j.1744-7429.2007.00271.x.

[ece371797-bib-0013] Crouzeilles, R. , M. Curran , M. S. Ferreira , D. B. Lindenmayer , C. E. V. Grelle , and J. M. Rey Benayas . 2016. “A Global Meta‐Analysis on the Ecological Drivers of Forest Restoration Success.” Nature Communications 7: 11666. 10.1038/ncomms11666. PMC4874030 27193756

[ece371797-bib-0014] Duan, W. J. , H. Ren , S. L. Fu , Q. F. Guo , and J. Wang . 2008. “Pathways and Determinants of Early Spontaneous Vegetation Succession in Degraded Lowland of South China.” Journal of Integrative Plant Biology 50, no. 2: 147–156. 10.1111/j.1744-7909.2007.00603.x. 18713436

[ece371797-bib-0015] Elmshagen, B. , G. Ludwig , S. P. Rushton , P. Helle , and H. Lindén . 2010. “Top Predators, Mesopredators and Their Prey: Interference Ecosystems Along Bioclimatic Productivity Gradients.” Journal of Animal Ecology 79, no. 4: 785–794. 10.1111/j.1365-2656.2010.01678.x. 20337755

[ece371797-bib-0016] Finnegan, S. P. , M. G. Gantchoff , J. E. Hill , et al. 2021. ““When the Felid's Away, the Mesocarnivores Play”: Seasonal Temporal Segregation in a Neotropical Carnivore Guild.” Mammalian Biology 101, no. 5: 631–638. 10.1007/s42991-021-00110-9.

[ece371797-bib-0017] Foster, V. C. , P. Sarmento , R. Sollmann , et al. 2013. “Jaguar and Puma Activity Patterns and Predator‐Prey Interactions in Four Brazilian Biomes.” Biotropica 45, no. 3: 373–379. 10.1111/btp.12021.

[ece371797-bib-0018] Grabowski, K. L. , E. M. Phillips , and K. M. Gaynor . 2024. “Limited Spatiotemporal Niche Partitioning Among Mesocarnivores in Gorongosa National Park, Mozambique.” Ecology and Evolution 14, no. 2: e10965. 10.1002/ece3.10965. 38371865 PMC10869889

[ece371797-bib-0019] Hardin, G. 1960. “The Competitive Exclusion Principle.” Science 131, no. 3409: 1292–1297. 10.1126/science.131.3409.1292. 14399717

[ece371797-bib-0020] Hendry, A. , Z. Amir , H. Decoeur , et al. 2023. “Marbled Cats in Southeast Asia: Are Diurnal and Semi‐Arboreal Felids at Greater Risk From Human Disturbances?” Ecosphere 14, no. 1: e4338. 10.1002/ecs2.4338.

[ece371797-bib-0021] Jennings, A. P. , and G. Veron . 2015. “Predicted Distributions, Niche Comparisons, and Conservation Status of the Spotted Linsang ( *Prionodon pardicolor* ) and Banded Linsang ( *Prionodon linsang* ).” Mammal Research 60, no. 2: 107–116. 10.1007/s13364-014-0204-y.

[ece371797-bib-0022] Kamler, J. F. , X. Inthapanya , A. Rasphone , et al. 2020. “Diet, Prey Selection, and Activity of Asian Golden Cats and Leopard Cats in Northern Laos.” Journal of Mammalogy 101, no. 5: 1267–1278. 10.1093/jmammal/gyaa113.

[ece371797-bib-0023] Kays, R. , B. S. Arbogast , M. Baker‐Whatton , et al. 2020. “An Empirical Evaluation of Camera Trap Study Design: How Many, How Long and When?” Methods in Ecology and Evolution 11, no. 6: 700–713. 10.1111/2041-210X.13370.

[ece371797-bib-0024] Lai, J. , W. Zhu , D. Cui , and L. Mao . 2023. “Extension of the *glmm.Hp* Package to Zero‐Inflated Generalized Linear Mixed Models and Multiple Regression.” Journal of Plant Ecology 16, no. 6: rtda0.38. 10.1093/jpe/rtad038.

[ece371797-bib-0025] Lai, J. , Y. Zou , S. Zhang , X. Zhang , and L. Mao . 2022. “Glmm.Hp: An r Package for Computing Individual Effect of Predictors in Generalized Linear Mixed Models.” Journal of Plant Ecology 15, no. 6: 1302–1307. 10.1093/jpe/rtac096.

[ece371797-bib-0026] Lau, M. W.‐N. , J. R. Fellowes , and B. P. L. Chan . 2010. “Carnivores (Mammalia: Carnivora) in South China: A Status Review With Notes on the Commercial Trade.” Mammal Review 40, no. 4: 247–292. 10.1111/j.1365-2907.2010.00163.x.

[ece371797-bib-0027] Lee, S. X. T. , Z. Amir , J. H. Moore , K. M. Gaynor , and M. S. Luskin . 2024. “Effects of Human Disturbances on Wildlife Behaviour and Consequences for Predator‐Prey Overlap in Southeast Asia.” Nature Communications 15, no. 1: 1521. 10.1038/s41467-024-45905-9. PMC10876642 38374248

[ece371797-bib-0028] Li, Y. , Z. Wu , L. Zhu , X. Huang , and J. Mo . 2024. “Innovative Reconstruction and Evaluation of Forest Refinement Datasets by Combining Multi‐Source Data: A Case Study of Guangdong Province.” Ecological Indicators 169: 112788. 10.1016/j.ecolind.2024.112788.

[ece371797-bib-0029] Manlick, P. J. , J. E. Woodford , B. Zuckerberg , and J. N. Pauli . 2017. “Niche Compression Intensifies Competition Between Reintroduced American Martens ( *Martes americana* ) and Fishers ( *Pekania pennanti* ).” Journal of Mammalogy 98, no. 3: 690–702. 10.1093/jmammal/gyx030.

[ece371797-bib-0030] Marinho, P. H. , C. R. Fonseca , P. Sarmento , C. Fonseca , and E. M. Venticinque . 2020. “Temporal Niche Overlap Among Mesocarnivores in a Caatinga Dry Forest.” European Journal of Wildlife Research 66, no. 2: 34. 10.1007/s10344-020-1371-6.

[ece371797-bib-0031] Mazerolle, M. 2006. “Improving Data Analysis in Herpetology: Using Akaike's Information Criterion (AIC) to Assess the Strength of Biological Hypotheses.” Amphibia‐Reptilia 27, no. 2: 169–180. 10.1163/156853806777239922.

[ece371797-bib-0032] Mengüllüoğlu, D. , and H. Ambarlı . 2019. “Assessing Caracal‐Prey Interactions by Spatial and Temporal Analyses.” European Journal of Wildlife Research 65, no. 4: 54. 10.1007/s10344-019-1294-2.

[ece371797-bib-0033] Nooten, S. S. , H. Korten , T. Schmitt , and Z. Karpati . 2024. “The Heat Is on: Reduced Detection of Floral Scents After Heatwaves in Bumblebees.” Proceedings of the Royal Society B: Biological Sciences 291, no. 2029: 20240352. 10.1098/rspb.2024.0352. PMC11349442 39191280

[ece371797-bib-0034] Nouvellet, P. , G. S. A. Rasmussen , D. W. Macdonald , and F. Courchamp . 2012. “Noisy Clocks and Silent Sunrises: Measurement Methods of Daily Activity Pattern.” Journal of Zoology 286, no. 3: 179–184. 10.1111/j.1469-7998.2011.00864.x.

[ece371797-bib-0035] Paine, R. T. 1966. “Food Web Complexity and Species Diversity.” American Naturalist 100, no. 910: 65–75. 10.1086/282400.

[ece371797-bib-0036] Pereira, R. , G. Matias , M. Santos‐Reis , and L. M. Rosalino . 2024. “Influence of Habitat Edges on Spatial and Spatio‐Temporal Occurrence Patterns of Mesocarnivores in Landscapes Dominated by Eucalyptus Plantations.” Forest Ecology and Management 572: 122257. 10.1016/j.foreco.2024.122257.

[ece371797-bib-0037] Pianka, E. R. 1974. “Niche Overlap and Diffuse Competition.” Proceedings of the National Academy of Sciences of the United States of America 71, no. 5: 2141–2145. 10.1073/pnas.71.5.2141. 4525324 PMC388403

[ece371797-bib-0038] Prugh, L. R. , C. J. Stoner , C. W. Epps , et al. 2009. “The Rise of the Mesopredator.” Bioscience 59, no. 9: 779–791. 10.1525/bio.2009.59.9.9.

[ece371797-bib-0039] Qian, G. , H. Sheng , and P. Wang . 1976. “Food Habits of *Melogale moschata* in Winter.” Chinese Journal of Zoology 21, no. 1: 37. 10.13859/j.cjz.1976.01.022.

[ece371797-bib-0040] Qin, Y. , and P. J. Nyhus . 2018. “Assessing Factors Influencing a Possible South China Tiger Reintroduction: A Survey of International Conservation Professionals.” Environmental Conservation 45, no. 1: 58–66. 10.1017/S0376892917000182.

[ece371797-bib-0041] R Core Team . 2024. R: A Language and Environment for Statistical Computing. R Foundation for Statistical Computing. https://www.R‐project.org/.

[ece371797-bib-0042] Ridout, M. S. , and M. Linkie . 2009. “Estimating Overlap of Daily Activity Patterns From Camera Trap Data.” Journal of Agricultural, Biological, and Environmental Statistics 14, no. 3: 322–337. 10.1198/jabes.2009.08038.

[ece371797-bib-0043] Ripple, W. J. , and R. L. Beschta . 2012. “Trophic Cascades in Yellowstone: The First 15 Years After Wolf Reintroduction.” Biological Conservation 145, no. 1: 205–213. 10.1016/j.biocon.2011.11.005.

[ece371797-bib-0044] Ripple, W. J. , J. A. Estes , R. L. Beschta , et al. 2014. “Status and Ecological Effects of the World's Largest Carnivores.” Science 343, no. 6167: 1241484. 10.1126/science.1241484. 24408439

[ece371797-bib-0045] Ritchie, E. G. , and C. N. Johnson . 2009. “Predator Interactions, Mesopredator Release and Biodiversity Conservation.” Ecology Letters 12, no. 9: 982–998. 10.1111/j.1461-0248.2009.01347.x. 19614756

[ece371797-bib-0046] Rodríguez‐Luna, C. R. , J. Servín , D. Valenzuela‐Galván , and R. List . 2024. “A Matter of Time Not of Co‐Occurrence: Temporal Partitioning Facilitates Coexistence Between Coyotes ( *Canis latrans* ) and Gray Foxes ( *Urocyon cinereoargenteus* ) in Temperate Forests of Mexico.” Mammalian Biology 104, no. 4: 363–377. 10.1007/s42991-024-00412-8.

[ece371797-bib-0047] Roemer, G. W. , M. E. Gompper , and B. Van Valkenburgh . 2009. “The Ecological Role of the Mammalian Mesocarnivore.” Bioscience 59, no. 2: 165–173. 10.1525/bio.2009.59.2.9.

[ece371797-bib-0048] Rowcliffe, J. M. , R. Kays , B. Kranstauber , C. Carbone , and P. A. Jansen . 2014. “Quantifying Levels of Animal Activity Using Camera Trap Data.” Methods in Ecology and Evolution 5, no. 11: 1170–1179. 10.1111/2041-210X.12278.

[ece371797-bib-0049] Schoener, T. W. 1974. “Resource Partitioning in Ecological Communities.” Science 185, no. 4145: 27–39. 10.1126/science.185.4145.27. 17779277

[ece371797-bib-0050] Silmi, M. , K. Putra , A. Amran , et al. 2021. “Activity and Ranging Behavior of Leopard Cats (*Prionailurus bengalensis*) in an Oil Palm Landscape.” Frontiers in Environmental Science 9: 651939. 10.3389/fenvs.2021.651939.

[ece371797-bib-0051] St‐Pierre, C. , J. P. Ouellet , and M. Crête . 2006. “Do Competitive Intraguild Interactions Affect Space and Habitat Use by Small Carnivores in a Forested Landscape?” Ecography 29, no. 4: 487–496. 10.1111/j.0906-7590.2006.04395.x.

[ece371797-bib-0052] Sun, Z. , H. Ren , V. Schaefer , et al. 2013. “Quantifying Ecological Memory During Forest Succession: A Case Study From Lower Subtropical Forest Ecosystems in South China.” Ecological Indicators 34: 192–203. 10.1016/j.ecolind.2013.05.010.

[ece371797-bib-0053] Teixeira, D. F. , A. J. Carpio , L. M. Rosalino , and D. Carniato . 2023. “Can Eucalyptus Plantations Influence the Distribution Range of Mesocarnivores?” Landscape Ecology 38: 3221–3235. 10.1007/s10980-023-01787-8.

[ece371797-bib-0054] Tian, J. , Q. Zou , M. Zhang , C. Hu , R. H. Khattak , and H. Su . 2022. “Spatial and Temporal Partitioning Are Not Distinct but Are Covariant for Facilitating Coexistence of Small and Medium‐Sized Carnivores in Southwestern China.” Global Ecology and Conservation 34: e02017. 10.1016/j.gecco.2022.e02017.

[ece371797-bib-0055] Tomassini, O. , A. Favilla , A. Aghemo , et al. 2024. “Wildfires Affect Mesocarnivores Habitat Use and Mammalian Predator‐Prey Relationships in a Mediterranean Ecosystem.” Acta Oecologica 123: 103986. 10.1016/j.actao.2024.103986.

[ece371797-bib-0056] Torretta, E. , M. Serafini , F. Puopolo , and L. Schenone . 2016. “Spatial and Temporal Adjustments Allowing the Coexistence Among Carnivores in Liguria (N‐W Italy).” Acta Ethologica 19, no. 2: 123–132. 10.1007/s10211-015-0231-y.

[ece371797-bib-0057] Treves, A. , and K. U. Karanth . 2003. “Human‐Carnivore Conflict and Perspectives on Carnivore Management Worldwide.” Conservation Biology 17, no. 6: 1491–1499. 10.1111/j.1523-1739.2003.00059.x.

[ece371797-bib-0058] Vilella, M. , M. Ferrandiz Rovira , and F. Sayol . 2020. “Coexistence of Predators in Time: Effects of Season and Prey Availability on Species Activity Within a Mediterranean Carnivore Guild.” Ecology and Evolution 10, no. 20: 11408–11422. 10.1002/ece3.6778. 33144974 PMC7593183

[ece371797-bib-0059] Villar, N. 2023. “Trophic Cascades Help Restore Vegetation.” Science 382: 516–517. 10.1126/science.adl0578. 37917711

[ece371797-bib-0060] Wallach, A. D. , I. Izhaki , J. D. Toms , W. J. Ripple , and U. Shanas . 2015. “What Is an Apex Predator?” Oikos 124, no. 11: 1453–1461. 10.1111/oik.01977.

[ece371797-bib-0061] Wang, H. , and T. K. Fuller . 2003. “Food Habits of Four Sympatric Carnivores in Southeastern China.” Mammalia 67, no. 4: 513–520. 10.1515/mamm-2003-0405.

[ece371797-bib-0062] Xiong, M. , D. Wang , H. Bu , et al. 2017. “Molecular Dietary Analysis of Two Sympatric Felids in the Mountains of Southwest China Biodiversity Hotspot and Conservation Implications.” Scientific Reports 7: 41909. 10.1038/srep41909. 28195150 PMC5307313

[ece371797-bib-0063] Ye, Z. , and M. Zhang . 2023. “Distribution of Annual Precipitation in Guangdong and Influence of Large‐Scale Topography.” Acta Scientiarum Naturalium Universitatis Sunyatseni 62, no. 4: 45–53. 10.13471/j.cnki.acta.snus.2022D079.

[ece371797-bib-0064] Zhang, L. , Y. P. Wang , Y. B. Zhou , et al. 2010. “Ranging and Activity Patterns of the Group‐Living Ferret Badger *Melogale moschata* in Central China.” Journal of Mammalogy 91, no. 1: 101–108. 10.1644/09-MAMM-A-049R.1.

[ece371797-bib-0065] Zhao, G. , H. Yang , B. Xie , Y. Gong , J. Ge , and L. Feng . 2020. “Spatio‐Temporal Coexistence of Sympatric Mesocarnivores With a Single Apex Carnivore in a Fine‐Scale Landscape.” Global Ecology and Conservation 21: e00897. 10.1016/j.gecco.2019.e00897.

