# Supplementary material for: Temporal, Spatial and Prey Niche Partitioning Reveals Coexistence Mechanism of Mesocarnivores in Guangdong Province, South China
Source: Ecol Evol. 2025 Jul 13;15(7):e71797. doi: 10.1002/ece3.71797 (PMC12256565; doi:10.1002/ece3.71797)
Supplement: Supplementary file 2 — Table S1. Camera trap deployment and number of used cameras in studied nature reserves. [file ECE3-15-e71797-s002.docx]

**Table S1** Camera trap deployment and number of used cameras in studied nature reserves

| **Name** | **Cameras Used / Cameras Deployed** | **Years** |
| --- | --- | --- |
| Cheba Ling National Nature Reserve | 170/191 | 2020, 2022 |
| Luokeng Crocodile Lizard National Nature Reserve | 38/56 | 2021 |
| Nanling National Nature Reserve | 19/24 | 2021, 2023 |
| Shimen Tai National Nature Reserve | 142/163 | 2019-2023 |
| Shixing Nanshan Provincial Nature Reserve | 46/55 | 2020-2022 |
| Qingzhang Mountain Provincial Nature Reserve | 22/31 | 2019-2022 |
| Qingxin Baiwan Provincial Nature Reserve | 60/80 | 2020, 2022-2023 |
| Renhua Gaoping Provincial Nature Reserve | 10/11 | 2019-2021 |
| Guangdong Qujiang Shaxi Provincial Nature Reserve | 69/70 | 2020-2021 |
| Lianzhou Tianxin Provincial Nature Reserve | 34/44 | 2023 |
| Wengyuan Qingyun Mountain Provincial Nature Reserve | 17/17 | 2020-2022 |
| Xinfeng Yunji Mountain Provincial Nature Reserve | 80/84 | 2019, 2022-2023 |
| Huaiji Da Chouding Provincial Nature Reserve | 39/47 | 2019-2020, 2022-2023 |
| Ruyuan Grand Canyon Provincial Nature Reserve | 37/40 | 2020-2021 |
| Lechang Dayao Mountain Provincial Nature Reserve | 29/48 | 2020, 2022 |
| Huaiji San Yue Provincial Nature Reserve | 57/57 | 2020-2022 |
| Huanan Tiger Provincial Nature Reserve in Northern Guangdong | 9/9 | 2022 |
| Liannan Bandong Provincial Nature Reserve | 69/70 | 2020-2022 |
| Liannan Giant Salamander Provincial Nature Reserve | 41/43 | 2020, 2022-2023 |
| Lianshan Bijia Mountain Provincial Nature Reserve | 36/42 | 2020-2023 |
| Total | 1024/1128 |  |
